# Therapeutic Effect of Lecigel, Cetiol^®^CC, Activonol-6, Activonol-M, 1,3-Propanediol, Soline, and Fucocert^®^ (LCAA-PSF) Treatment on Imiquimod-Induced Psoriasis-like Skin in Mice

**DOI:** 10.3390/ijms25147720

**Published:** 2024-07-14

**Authors:** Chih-Ching Li, Chih-Chien Lin, Chun-Yi Lee, Meei-Ling Sheu, Yi-Ching Tsai, Chia-Yun Tsai, Hao-Ting Wu, Ren-Jang Wu, De-Wei Lai

**Affiliations:** 1 Department of Applied Chemistry, Providence University, 200, Sec. 7, Taiwan Boulevard, Shalu Dist., Taichung 43301, Taiwan; chingching3244@gmail.com; 2 Department of Pediatrics, Chang Bing Show Chwan Memorial Hospital, No. 6, Lugong Rd. Lugang Township, Changhua 505029, Taiwan; lee821083@gmail.com; 3 Department of Cosmetic Science, Providence University, 200, Sec. 7, Taiwan Boulevard, Shalu Dist., Taichung 43301, Taiwan; chchlin@pu.edu.tw; 4 Department of Post-Baccalaureate Medicine, College of Medicine, National Chung Hsing University, Taichung 40227, Taiwan; 5 Institute of Biomedical Sciences, National Chung Hsing University, Taichung 40227, Taiwan; mlsheu@nchu.edu.tw; 6 Department of Medical Research, Taichung Veterans General Hospital, Taichung 40210, Taiwan; 7 Rong Hsing Research Center for Translational Medicine, National Chung Hsing University, Taichung 40227, Taiwan; 8 Immunomedicine Group, Department of Molecular Biology and Cell Research, Chang Bing Show Chwan Memorial Hospital, Changhua 505029, Taiwan; yctsai1228@gmail.com; 9 Experimental Animal Center, Department of Molecular Biology and Cell Research, Chang Bing Show Chwan Memorial Hospital, Changhua 505029, Taiwan; n11232147@gmail.com (C.-Y.T.); j0903504723@gmail.com (H.-T.W.); 10 Department of Pharmacy and Master Program, Tajen University, Pingtung 907101, Taiwan

**Keywords:** 1,3-Propanediol, Soline, Fucocert^®^, imiquimod, psoriasis

## Abstract

The individual ingredients of 1,3-Propanediol, Soline, and Fucocert^®^ (PSF) are often used as cosmetic formulations in skin care. In addition, the mixture of Lecigel, Cetiol^®^CC, Activonol-6, and Activonol-M (LCAA) is often used as a cosmetic base. However, whether the combination of LCAA with PSF (LCAA-PSF) exerts a therapeutic effect on psoriasis remains unclear. In this study, mice induced with imiquimod (IMQ) were divided into three groups and administered 100 mg/day of LCAA, 100 mg/day of LCAA-PSF, or Vaseline on the dorsal skin of each mouse. Weight-matched mice treated with Vaseline alone were used as controls. Hematoxylin and eosin (H&E) staining and enzyme-linked immunosorbent assay(ELISA) were used to assess tissue morphology and inflammatory cytokines. RNA sequencing analysis was used to predict the mechanism underlying the action of LCAA-PSF against psoriasis, while immunohistochemical analysis validation was used to identify pertinent molecular pathways. The results demonstrated that LCAA-PSF alleviated IMQ-induced keratinocyte differentiation/ proliferation bydecreasingthe serum levels of inflammatory cytokines such as IL-6, TNF-α, IL-23, and IL-17A and the epidermisof TGFβ, Ki67, CK5/6, and VEGF expression, which is associated with angiogenesis and keratinocyte differentiation/ proliferation. These findings highlight the antipsoriatic activity of LCAA-PSF in a psoriasis-like mouse model and suggest this may occurvia the inhibition of inflammatory factor secretionand the TGFβ-related signal pathway.

## 1. Introduction

Psoriasis, a chronic immune-mediated inflammatory skin disease, has a global prevalence of 0.14–5.32% [1]. The diverse clinical manifestations of psoriasisare characterized by keratinocyte differentiation and excessive proliferation, well-defined erythematous, and silvery-white dry scales localized in one or multiple sites [2]. Psoriasis is treated using several conventional methods, including topical, oral, and biological treatments [3]. Topical treatments are the mainstay of treatment for mild-to-moderate psoriasis. However, topical application may produce adverse skin irritation and burning, leading to poor patient compliance. Oral treatment is mainly used when topical treatment fails to improve symptoms. However, oral treatment is less effective compared to topical treatment, and its side effects are more marked, resulting in lower acceptance [4,5]. Therefore, the development of novel treatment strategies, such as customized lotions, is crucial to improve the quality of life of patients with psoriasis.

Emulsion forms provide pharmaceutical formulations for the treatment of skin conditions and for skin care generally. Their primary objective is to enhance solubility, skin penetration, and topical penetration with the required drug loading [6]. 1,3-Propanediol has high moisturizing ability and solubility and improves hydration and skin barrier function [7,8]. Similarly, Soline (Helianthus Annuus seed oil unsaponifiables) can promote epidermal lipid regeneration and regulate its composition, strengthen intercellular interstitial bonding, and improve skin hydration [9]. Furthermore, it is mainly composed of tocopherols, plant sterols, alcohols, hydrocarbons, and phenolics, which have anti-inflammatory effects [10]. Fucocert^®^ is composed of three sequential sugars (L-fucose, D-galactose, and galacturonic acid) and inhibits the activation of MMP-2 and MMP-9 precursors [11]. Owing to its moisturizing, self-emulsifying properties, it is commonly used in skin-care products and helps to form a protective film over wounds [12]. Here, these three components were mixed in appropriate proportions to obtain PSF, which was then combined with the cosmetic base of Lecigel, Cetiol^®^CC, Activonol-6, and Activonol-M (LCAA) to produce LCAA-PSF. Although each of these components, namely LCAA and PSF are known to exert beneficial effects, it is unclear whether LCAA-PSF exerts a therapeutic effect on psoriasis.

The hyperproliferation and impaired differentiation of keratinocytes are typical histopathological features of psoriasis. Psoriasis progression is initiated by the activation of pro-inflammatory cytokines via the upregulation of keratinocytes and immune cells [13,14]. After the abnormal activation of dendritic cells is induced by IL-23,inflammatory cytokines, such as IL-17A, IL-22, TNF-α, and IL-6,are produced [15]. The IL-23/Th17 axis is thought to be closely related to the onset and maintenance of psoriasis. These inflammatory factors promote keratinocyte proliferation, among other symptoms of psoriasis. Imiquimod (IMQ) can exacerbate psoriasis at the site of administration, and as a systemic response [16,17]. IMQ application to mouse skin results in immune cell influx, changes in reactive dendritic cells, secretion inflammatory cytokines, and epidermal hyperplasia [18]. These effects manifest as dermatitis with symptoms identical to those of human psoriasis, thereby providing a useful model of human psoriasis to determine the efficacy of LCAA-PSF in IMQ-induced psoriasis treatment and clarify the mechanisms underlying psoriasis pathogenesis blockage.

## 2. Results

### 2.1. LCAA-PSF Ameliorated IMQ-Induced Psoriatic Skin Lesions in Mice

**Figure 1:**
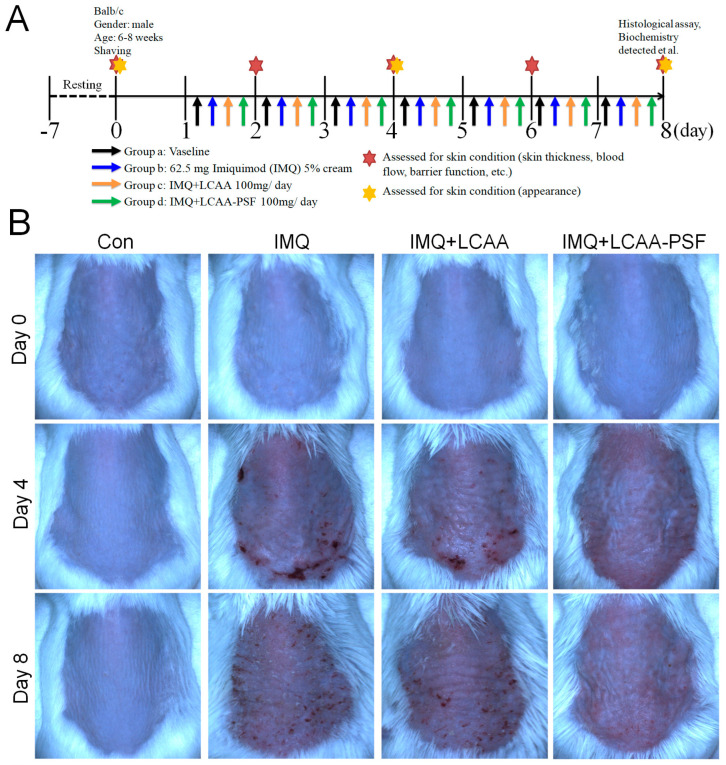

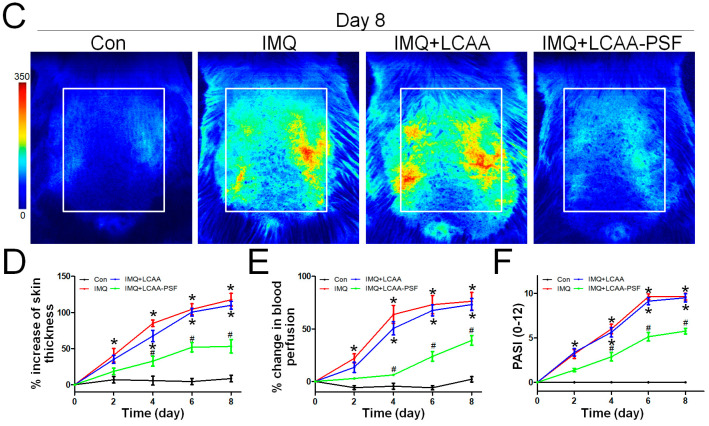
**LCAA-PSF attenuates IMQ-induced mouse psoriatic lesion.** (**A**) Flow chart of this experiment. (**B**) The backskin photos of mice were taken at days 0, 4, 8. (**C**) Representative images of blood perfusion changes on dorsal skin induced by topical application of Vaseline or IMQ at day 8. (**D**) Percent increase inback skin thickness in all groups compared to day 0 baseline values, n = 8/group. (**E**) Percent change inblood perfusion in dorsal skin in all groups compared to day 0 baseline values, n = 5/group. (**F**) PASI scores in all groups of mice were evaluated at days 0, 2, 4, 6, 8 and the statistical difference between all groups at 8th day was indicated, n = 8/group. Data are mean ± SEM. * *p* < 0.05 IMQ vs. Con group and IMQ+LCAA vs. Con group, # *p* < 0.05 IMQ vs. IMQ+LCAA-PSF group, based on repeated measures two-way ANOVA followed by Bonferroni’s post hoc test.

To investigate whether LCAA-PSF can ameliorate psoriatic skin lesions, we evaluated IMQ-induced psoriasis-like mice models with andwithout LCAA-PSF treatment. A schematic representation of animal experimentation is provided in the form of a flow chart in Figure 1A. IMQ 5% cream was applied to the exposed skin on the shaved backs of Balb/c mice for seven consecutive days with andwithout LCAA-PSF (100 mg/day) and LCAA (100 mg/day). Subsequently, the IMQ-treated mice developed typical psoriasis-like inflammatory responses on their skin, such as erythema, scaling, and thickening, compared to the control mice group at day 8. However, the administration of LCAA-PSF significantly ameliorated these skin lesions throughout the IMQ-induced period, while the administration of LCAA had no affect (Figure 1B). Furthermore, on day 8, the IMQ group showed elevated microcirculation in the dorsal skin compared to the control group, represented by white areas on images obtained using a LASCA laser Speckle instrument. Nevertheless, the IMQ+LCAA-PSF group showed a significantly reduced dorsal skin blood flow compared to the IMQ group, while the IMQ+LCAA group showed no significant difference (Figure 1C). The application of IMQ 5% cream significantly increased the thickness of the dorsal skin in the IMQ group (117.7 ± 8.8%)compared to the control mice (8.5 ± 4.5%) (*p* < 0.05) on day 8. In the IMQ+LCAA-PSF group, this was significantly reduced compared to the IMQ group (IMQ+LCAA-PSF group: 53.2 ± 9.2% vs. IMQ group: 117.7 ± 8.8%) (*p* < 0.05), while the IMQ+LCAA group showed no significant difference (IMQ+LCAA group: 110.0 ± 5.3% vs. IMQ group: 117.7 ± 8.8%) (*p* > 0.05) (Figure 1D). A significant change in dorsal skin blood perfusion was observed in the IMQ group compared to the control group on day 8 (IMQ group: 76.3 ± 8.4% vs. Con group: 2.1 ± 2.5%) (*p* < 0.05), which approached the maximal response (63.8 ± 8.5%) on day 4. However, administration in the IMQ+LCAA-PSF group notably decreased dorsal skin blood perfusion compared to the IMQ group (IMQ+LCAA-PSF group: 39.1 ± 4.6% vs. IMQ group: 76.3 ± 8.4%)(*p* < 0.05). No significant difference was observed in terms of skin blood perfusion between the IMQ+LCAA and IMQ groups (IMQ+LCAA group: 73.2 ± 5.7% vs. IMQ group: 76.3 ± 8.4%) (*p* > 0.05) (Figure 1E). The administration of LCAA-PSF notably alleviated the severity of IMQ-induced psoriasis compared to the IMQ model group, according to the scores of erythema, thickness, and cumulative score on day 8 (IMQ+LCAA-PSF group: 5.7 ± 0.3 vs. IMQ group: 9.6 ± 0.3) (*p* < 0.05) (Figure 1F). Taken together, LCAA-PSF was found to alleviate psoriatic skin lesions compared with the IMQ-induced model.

### 2.2. LCAA-PSF Treatment Comprehensively Improved the Dorsal Skin Condition in the IMQ-Induced Psoriasis Model

**Figure 2:**
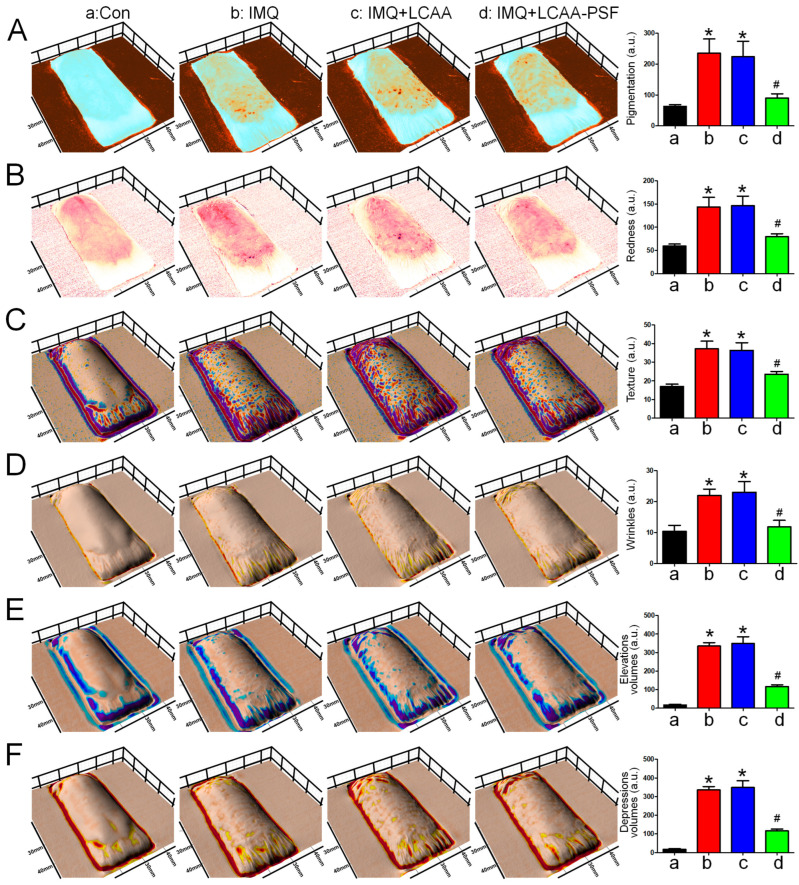
**Three-dimensionalskin imaging of an area of LCAA-PSF treatment on the dorsal skin of IMQ-induced at day 8.** (**A**) Melanin view showing areas of pigmentation. (**B**) Redness. (**C**) Texture. (**D**) Wrinkles. (**E**) Elevation volumes. (**F**) Depression volumes. Quantification is displayed in the right panel, respectively. Data are mean ± SEM. * *p* < 0.05 IMQ vs. Con group and IMQ+LCAA vs. Con group, # *p* < 0.05 IMQ vs. IMQ+LCAA-PSF group. a: control group; b: IMQ group; c: IMQ+LCAA group; d: IMQ+LCAA-PSF. Statistical analysis was performed by one-way ANOVA and Tukey post hoc tests.

To comprehensively evaluate the effects of LCAA-PSF on changes in the skin of IMQ-induced psoriasis model mice, the levels of pigmentation, redness, texture, wrinkles, and skin elevation and depression volumes were monitored using Antera 3D. The resulting images revealed that the IMQ group exhibited significantly increased pigmentation, redness, texture, wrinkles, and elevation and depression volumes. After treatment with LCAA-PSF, the levels of pigmentation, redness, texture, wrinkles, and elevation and depression volumes wereeffectively reduced (Figure 2A–E). Furthermore, the elevation and depression volumes were reduced by approximately 2- to 3-fold, with the skin becoming smoother. However, the results in the IMQ+LCAA group were equivalent to those of the IMQ group. These findings imply that LCAA-PSF may be effective in managing multifaceted skin conditions during the development of IMQ-induced psoriasis pathology.

### 2.3. LCAA-PSF Attenuated Tissue Pathology of Psoriasis

**Figure 3:**
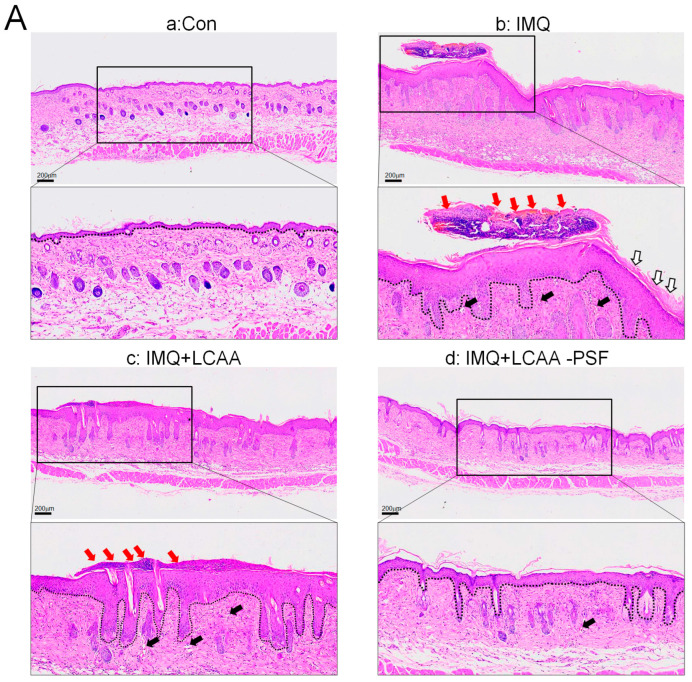

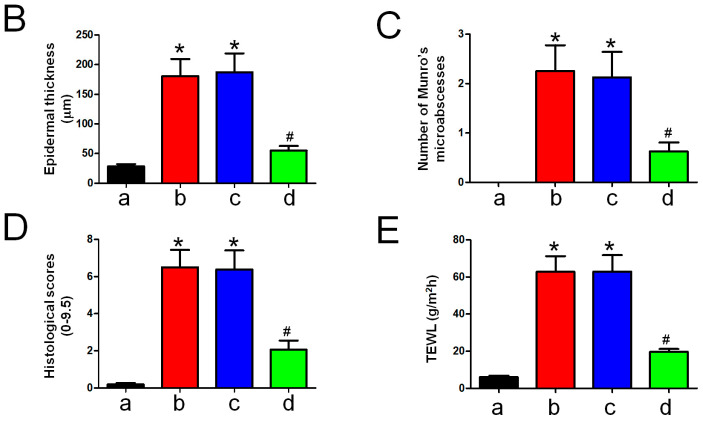
**Histological alterations following LCAA-PSF treatment on the dorsal skin of IMQ-induced mice at day 8.** (**A**) H&E staining of the dorsal skinat 20× and insets box 40× magnification. Red arrow: Munro’s microabscesses; black arrow: dilated capillaries; white arrow: hyperkeratosis and parakeratosis; the dotted line in each section highlights the epidermal–dermal junction. (**B**) Thickness of epidermal layer. (C) Number of Munro’s microabscesses. (**D**) Histopathological score. (**E**) TEWL measurement. Data are mean ± SEM. * *p* < 0.01 IMQ vs. Con group and IMQ+LCAA vs. Con group, # *p* < 0.01 IMQ vs. IMQ+LCAA-PSF group. a: control group; b: IMQ group; c: IMQ+LCAA group; d: IMQ+LCAA-PSF. Statistical analysis was performed by one-way ANOVA and Tukey post hoc tests.

H&E staining revealed that the epidermal layer in the IMQ group was thickened, and numerous Munro’s microabscesses were observed in the dermis and epidermis (red arrow). After treatment with LCAA-PSF, the thickness of the epidermal layer was effectively reduced, the stratum corneum became smooth, and the number of Munro’s microabscesses decreased (Figure 3A). The epidermal thickness in the IMQ+LCAA-PSF group (55.2 ± 7.7 μm) was found to decrease compared with the psoriasiform lesion in the IMQ group (180.8 ± 28.7 μm) (Figure 3B). Moreover, the number of Munro’s microabscesses throughout the sections in the IMQ+LCAA-PSF group counts (0.6 ± 0.2/field (200×)) was found to decrease compared with thatin the IMQ group (2.3 ± 0.5/ field (200×)). The Baker scoring system (keratinocytes hyperproliferation, hyperkeratosis, parakeratosis, and Munro’s microabscesses) was used to evaluate the histopathological scoring of the severity of inflammation in all experimental groups. As a result, the histology of LCAA-PSF-treated skin exhibited a significant improvement with respect to inflammatory symptoms. The total histological scores showed that the IMQ+LCAA-PSF group had a significantly lower score than the IMQ group (Figure 3D). TEWL, an indicator of skin barrier function, was found to exhibit a 10-fold increase in IMQ-treated skin compared to the control group. However, a 3-fold decrease in this indicator was observed in the IMQ+LCAA-PSF group compared to the IMQ group. These results are indicative of barrier dysfunction in the psoriasiform skin of the IMQ group. By contrast, LCAA-PSF treatment was found to attenuate pathological development.

### 2.4. LCAA-PSF Decreased the Inflammatory Response Both Locally and Systemically inthe IMQ-Induced Psoriasis Model

**Figure 4:**
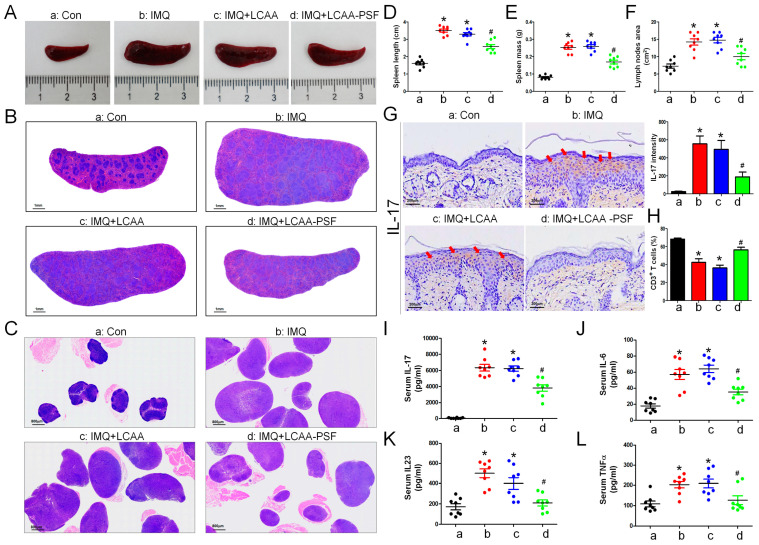
**LCAA-PSF suppressed IMQ-induced inflammatory cytokines releasing in skin and systemic inflammation at day 8.** (**A**) The gross images of mouse spleen were represented by digital camera. (**B**) H&E staining of the spleenat 5× magnification, (**C**) and skin draining lymph nodes at 5× magnification. (**D**) Quantification of spleen length, (**E**) mass, and (**F**) area of lymph nodes (cm^2^). (**G**) Expression of IL-17 from dorsal skin samples was detected by IHC stainingat 60× magnification (red arrow indicates staining site), and quantitative analysis of expression level of IL-17. (**H**) Spleen cells were analyzed for the percentage of T cells (CD3+) by flow cytometry. The serum levels of the inflammatory cytokines, including (**I**) IL-17A, (**J**) IL-6, (**K**) IL-23, (**L**) TNFα. Data are mean ± SEM. * *p* < 0.01 IMQ vs. Con group and IMQ+LCAA vs. Con group, # *p* < 0.01 IMQ vs. IMQ+LCAA-PSF group. a: control group; b: IMQ group; c: IMQ+LCAA group; d: IMQ+LCAA-PSF. Statistical analysis was performed by one-way ANOVA and Tukey post hoc tests.

Macroscope imaging (Figure 4A) and H&E staining (Figure 4B) of the spleen and skin draining lymph nodes (Figure 4C) showed an increase in the mass and length with the progression of psoriasis in the IMQ group. The length and mass of the spleen was found to increase significantly on day 8 compared with the control group, while that of the IMQ+LCAA-PSF group decreased significantly compared with that of the IMQ group (Figure 4D,E). Furthermore, a similar trend was observed in terms of the area of the lymph nodes. The results showed a 2-fold increase in the size of the lymph nodes in the IMQ group compared with the control group, while this was significantly reversed in the IMQ+LCAA-PSF group (Figure 4F). The activation of the lymph nodes is known to be related to the severity of psoriasis. The pro-inflammatory cytokine IL-17 was detected in the epidermis by immunohistochemistry(IHC) staining. The results showed that IL-17 was overexpressed in the IMQ group, while its expression was significantly decreased in the IMQ+LCAA-PSF (Figure 4G). Furthermore, the T cell population of the spleens of mice was determined by flow cytometry. The percentage of T cells (CD3+) was decreased in the IMQ group compared with the control group, whereas its effect was reversed in the LCAA-PSF group (Figure 4H) (*p* < 0.01). Additionally, the effects of LCAA-PSF on serum inflammatory cytokines were detected using enzyme-linked immunosorbent assay (ELISA). The results showed that the IL-17A, IL-6, IL-23, and TNF-α levels increased significantly in IMQ-induced mice with psoriasis. The levels of these cytokines also significantly decreased in the LCAA-PSF group compared with the IMQ group (Figure 4I–L) (*p* < 0.01). These results suggest that LCAA-PSF reduced the expression of proinflammatory cytokinesand regulated immune homeostasis in the IMQ-induced psoriasis mouse model.

### 2.5. Molecular Mechanism of LCAA-PSF in Regulating Psoriasis

**Figure 5:**
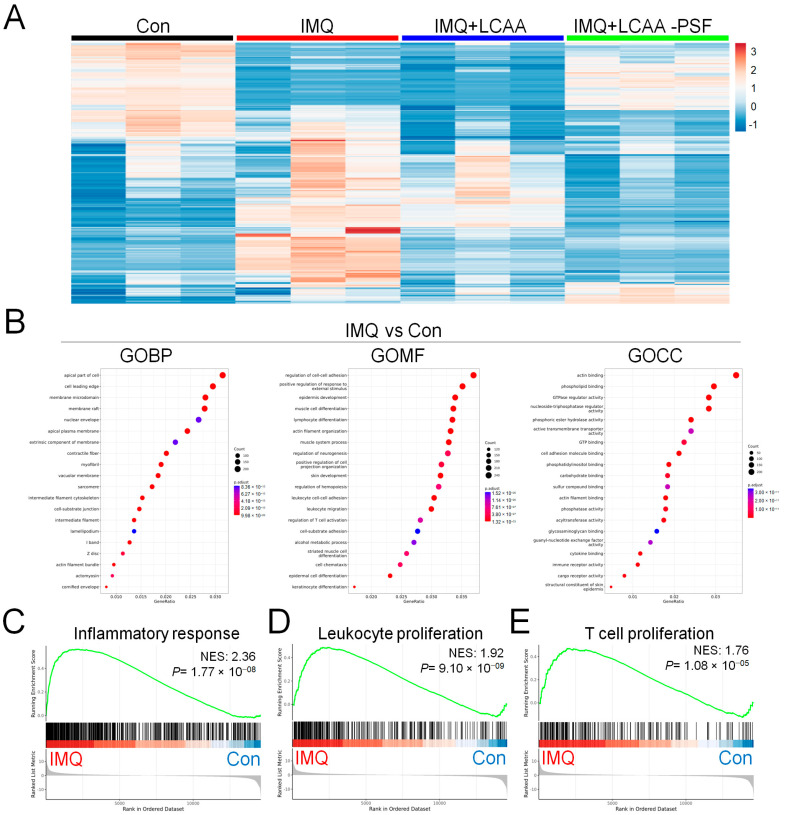

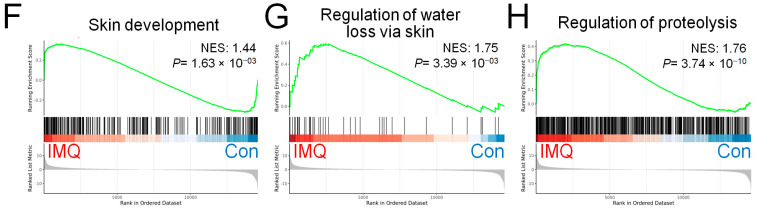
**Genomics of Con, IMQ, IMQ+LCAA and IMQ+LCAA-PSF groups from dorsal skin samples.** Transcriptomic profiles of Con, IMQ, IMQ+LCAA and IMQ+LCAA-PSF groups dorsal skin samples were determined by RNA-seq. (**A**) Hierarchical clustering heatmap depicting individual samples showed differential gene expression patterns between Con, IMQ, IMQ+LCAA and IMQ+LCAA-PSF groups. The colors at the top of the heat map indicate the following: black indicates the Con group; red indicates the IMQ group; blue indicates the IMQ+LCAA group; and green indicates the IMQ+LCAA-PSF group. The map with color in the upper and outer quadrants represents differential gene expression, with blue indicating a relative decrease and red indicating a relative increase in abundance. Selected top 290 gene represents (n = 3 per group). Gene ontology (GO) themes enriched in IMQ versus Con and IMQ+LCAA-PSF versus IMQ dorsal skin tissue in mice. IMQ versus Con resulting (**B**) the top 20 gene ontology biological process (BP) terms with the highest statistical significance. The vertical axis is the GOBP terms, and the horizontal axis is the number of genes. The top 20 gene ontology molecular function (MF) terms with the highest statistical significance. The vertical axis is the GOMF terms, and the horizontal axis is the number of genes. The top 20 gene ontology cellular component (CC) terms with the highest statistical significance. The vertical axis is the GOCC terms, and the horizontal axis is the number of genes. GOBP database (**C**) inflammatory response pathway, (**D**) leukocyte proliferation pathway, (**E**) T cell proliferation pathway, (**F**) skin development, (**G**) regulation of water loss via skin, (**H**) regulation of proteolysis GSEA signatures in IMQ were compared with Con. NES and P value were obtained by GSEA in the HALLMARK database.

To investigate the mechanism by which LCAA-PSF regulates IMQ-induced psoriasis, we performed RNA sequencing analysis using the dorsal skin tissues obtained from the control, IMQ, IMQ+LCAA, and IMQ+LCAA-PSF groups (Figure 5A). As a result, the distribution patterns of enriched gene expression volcano plots in the IMQ group vs. Con group, IMQ+LCAA-PSF group vs. IMQ group, IMQ+LCAA group vs. IMQ group, and IMQ+LCAA group vs. IMQ+LCAA-PSF group were evaluated using log-transformed gene abundance ratios (Supplementary Figure S1A–D). As a result, DEGs were observed in the IMQ group vs. Con group ([894 upregulated, 981 downregulated; FC ≥ 2.0 or ≤−2.0; *p* < 0.05] vs. IMQ+LCAA-PSF group vs. IMQ group [345 upregulated, 241 downregulated; FC ≥ 2.0 or ≤−2.0; *p* < 0.05]). Furthermore, the differential genes of the two groups were combined, and based on the resulting Venn diagram,251 of the common intersection genes were analyzed by gene ontology (Supplementary Figure S1E). Analysis of the biological process (GOBP) of the differential genes in the IMQ group vs. Con group showed significant differential changes in various pathways related to apical part of cell, cell leading edge, and membrane microdomain. Similarly, analysis of the molecular function (GOMF) of the differential genes highlighted the regulation of cell–cell adhesion, epidermis development, and regulation of T cell activation. Analysis of the cellular components (GOCC) of the differential genes highlighted cell adhesion molecule binding, cytokine binding, and immune receptor activity (Figure 5B). Finally, transcriptomic analysis confirmed enrichment in a defined inflammatory response, leukocyte proliferation, T cell proliferation, skin development, regulation of water loss via the skin, and regulation of proteolysis in the IMQ group compared with the Con group (Figure 5C–H).

### 2.6. Molecular Mechanism Underlying the Attenuation of Keratinocyte Differentiation/Proliferationand Angiogenesis by LCAA-PSF

**Figure 6:**
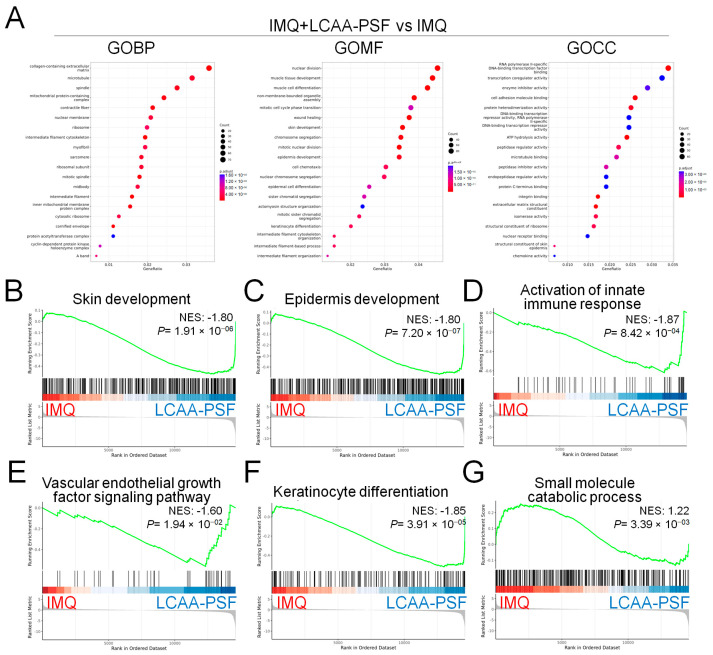

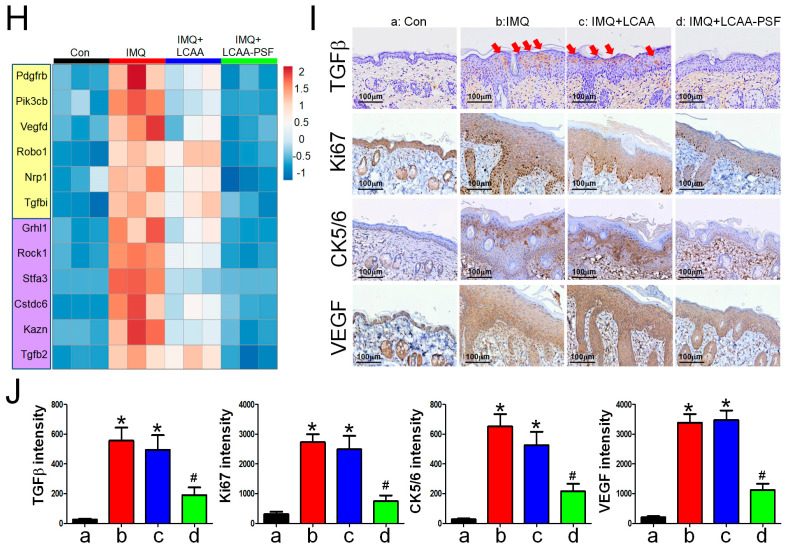
**Mechanistical regulation of LCAA-PSF in the IMQ-induced mouse psoriasis.** IMQ+LCAA-PSF versus IMQ group shows (**A**) the top 20 gene ontology biological process (BP) terms with the highest statistical significance. The vertical axis is the GOBP terms, and the horizontal axis is the number of genes. The top 20 gene ontology molecular function (MF) terms with the highest statistical significance. The vertical axis is the GOMF terms, and the horizontal axis is the number of genes. The top 20 gene ontology cellular component (CC) terms with the highest statistical significance. The vertical axis is the GOCC terms, and the horizontal axis is the number of genes. GOBP database (**B**) skin development pathway, (**C**) epidermis development pathway, (**D**) activation of innate immune response pathway, (**E**) vascular endothelial growth factor signaling pathway, (**F**) keratinocyte differentiation, (**G**) small molecule catabolic process GSEA signatures in IMQ+LCAA-PSF were compared with IMQ. NES and *p* value were obtained by GSEA in the HALLMARK database. (**H**) Heatmap representing the relative expression levels of selected the top 6 genes (Pdgfrb, Pik3cb, Vegfd, Robo1, Nrp1 and Tgfbi in yellow background indicated angiogenesis pathway; Grhl1, Rock1, Stfa3, Cstdc6, Kazn and Tgfb2 in purple background indicated keratinocyte differentiation pathway, respectively) expressed from GOBP database with the highest statistical significance. (**I**) Expression of TGFβ, Ki67, CK 5/6 and VEGF from dorsal skin samples was detected by IHC staining at 60× magnification. (**J**) Quantitative analysis of expression level of TGFβ, Ki67, CK 5/6 and VEGF. Data are mean ± SEM. * *p* < 0.01 IMQ vs. Con group and IMQ+LCAA vs. Con group, # *p* < 0.01 IMQ vs. IMQ+LCAA-PSF group. a: control group; b: IMQ group; c: IMQ+LCAA group; d: IMQ+LCAA-PSF. Statistical analysis was performed by one-way ANOVA and Tukey post hoc tests.

As depicted in the Venn diagram, the expression of 116 DEGs increased in the IMQ group and decreased in the IMQ+LCAA-PSF group (Supplementary Figure S1F), which wassubsequently further analyzed by gene ontology. The analysis of the biological process (GOBP) of the differential genes in the IMQ+LCAA-PSF group vs. IMQ group showed significant differential changes in various pathways related to collagen-containing extracellular matrix, microtubules, and spindle. Furthermore, the analysis of the molecular function (GOMF) of the differential genes highlighted skin development, epidermis development, and keratinocyte differentiation. In addition, the analysis of the cellular components (GOCC) of the differential genes highlighted enzyme inhibitor activity, peptidase regulator activity, and structural constituent of skin epidermis (Figure 6A). Lastly, transcriptomic analysis confirmed less enrichment in a defined skin development, epidermis development, the activation of the innate immune response, vascular endothelial growth factor signaling pathway, and keratinocyte differentiation in the IMQ+LCAA-PSF groupcompared with the IMQ group (Figure 6B–F). On the other hand, transcriptomic enrichment was observed in a defined small molecule catabolic process in the IMQ+LCAA-PSF group compared with the IMQ group (Figure 6G). Furthermore, the top 12 significantly differentiated genes were selected from 116 genes, including six genes related to the vascular endothelial growth factor signaling pathway (Pdgfrb, Pik3cb, Vegfd, Robo1, Nrp1, and Tgfbi) (Figure 6H, yellow background) and six genes related to the keratinocyte differentiation pathway(Grhl1, Rock1, Stfa3, Cstdc6, Kazn, and Tgfb2) (Figure 6H, purple background). The TGFβ signaling pathway was mostly associated with keratinocyte differentiation and angiogenesis, which may play a key role in the development of psoriasis pathology. Given the consistent enrichment of genes related to the TGFβ signaling pathways observed in the results of our transcriptomics analyses, we further examined the expression levels of TGFβ in the epidermis. Compared to the control group, the IMQ group displayed notably elevated protein expression levels of TGFβ. However, by contrast, the experimental group of mice treated with LCAA-PSF exhibited a substantial decrease in the expression of TGFβ (Figure 6I). Moreover, psoriasis induced by IMQ manifests through the excessive proliferation and aberrant differentiation of keratinocytes as well as enhanced angiogenesis. These pathological changes are typified by elevated expressions of Ki67,CK5/6 and VEGF, respectively, serving as biomarkers for cellular proliferation/ keratinocyte differentiation and vascular growth. As shown in Figure 6I, compared to the control group, the IMQ group displayed notably elevated protein expression levels of Ki67, CK5/6 and VEGF. Conversely, the experimental group of mice treated with LCAA-PSF exhibited a substantial decrease in the expression of Ki67, CK5/6 and VEGF. The quantitative expressions of TGFβ, Ki67, CK5/6 and VEGF in different groups are shown in Figure 6J. These results suggest that LCAA-PSF reduced the excessive proliferation and aberrant differentiation of keratinocytes in the IMQ-induced psoriasis mouse model.

## 3. Discussion

Psoriasis is a complex disease caused by multiple factors whose precise pathogenesis remains unclear. Previous studies suggested that keratinocyte hyperproliferation and abnormal differentiation may play a key role in the development and maintenance of psoriasis [19]. The IL-23/Th17 axis is thought to be closely related to the pathogenesis of psoriasis. Activated by IL-23 and released from dendritic cells and macrophages, Th17 and T cells in the psoriatic dermis secrete inflammatory cytokines, including IL-23, IL-17A, TNF-α, and IL-6 [20]. These inflammatory cytokines act on keratinocytes, leading to epidermal hyperplasia, hyperkeratosis, and other typical pathological changes inpsoriasis [21]. Particularly in the inflammatory microenvironment of the skin, keratinocytes produce more IL-23, chemokines, and other inflammatory factors, which results in the formation of the IL-23/Th17 axis, which, in turn, intensifies the inflammatory response and drives keratinocyte hyperproliferation in psoriasis [22,23]. In the present study, LCAA-PSF was found to effectively slow down the inflammatory cytokine secretion of IMQ-induced psoriasis, including the expression of IL-23, IL-17A, TNF-α, and IL-6 in the serum and the expression of IL-17, Ki67, CK5/6 and VEGF in the affected skin area. This was consistent with the findings reported in the literature. These results imply that LCAA-PSF can reduce the proliferation of keratinocytes and alleviate the symptoms of psoriatic dermatitis by reducing the secretion of inflammatory factors.

Transforming growth factor beta (TGFβ) is a pluripotent cytokine that regulates cell growth and differentiation. Three isoforms of TGFβ (TGFβ1, 2, and 3) were documented in human tissues. In most cell types, all three forms have similar biological activities; however, TGFβ1 is the main isomer in most tissues, including in the skin [24]. In non-pathological skin tissue, TGFβ has been shown to inhibit keratinocyte growth but stimulate fibroblast growth and regulator T cells [25]. However, TGFβ1 also exerts pro-inflammatory effects. For example, in the skin, TGFβ1 is required for the development and maturation of Langerhans cells. Furthermore, TGFβ1 is required for the differentiation of naive CD4+ T cells into pro-inflammatory interleukin-17-producing T helper cells (Th17 cells), and TGFβ1 transgenic mice exhibit marked epidermal hyperplasia, accompanied by inflammatory cell infiltration and neovascularization, mimicking the characteristics of human psoriasis [26]. In agreement with previous studies, IMQ application increased keratinocyte differentiation and vascular endothelial growth factor signaling pathway cascades, which may be related to TGFβ expression in the dorsal skin. Molecularly, LCAA-PSF treatment reduced the expression of TGFβ and inhibited both keratinocyte differentiation and the vascular endothelial growth factor signaling pathway in the IMQ-induced mice. In addition, LCAA-PSF may be used as a target for the treatment of IMQ-induced psoriasis through its potential anti-TGFβ. In the future, the specific mechanism throughwhich LCAA-PSF intervenes to interfere with TGFβ-induced skin inflammation will need to be further analyzed, such as in terms of its involvement in Smad or non-Smad pathways.

Psoriatic lesions resulting from proteolytic activity can persist for long periods of time in the upper epidermis, accompanied by temporary acanthosis and inflammatory cells around the superficial blood vessels, and may even result in typical Koebner phenomena [27]. Previous studies reported that protease inhibitors regulate various biological processes and prevent host tissue damage. In particular, the inhibition of proteases is known to be of clinical value for the treatment of various chronic inflammation diseases [28]. Cysteine protease inhibitors have a high specificity, selectivity, and affinity for target proteases and are effective immunomodulators [29]. In the present study, the results of RNA sequencing and gene set enrichment analysis showed that IMQ vs. Con exhibited significantly increased regulation of proteolysis, which can correspond to IMQ+LCA-PSF vs. IMQ, in which significantly increased small molecule catabolic processes were observed, including Slpi (secretory leukocyte peptidase inhibitor), Wfdc3, Wfdc5, Mt3, and Cyp4f18. This was consistent with previous findings reported in the literature. These results suggest that LCAA-PSF may potentially regulate the proteolytic activity in the skin by activating a variety of small molecule proteins, thereby affecting immune homeostasis.

Taken together, the findings indicate that LCAA-PSF primarily targeted keratinocytes in vivo by inducing the secretion of “IL-23/Th17 axis”-related inflammatory factors, thereby reducing the hyperproliferation of keratinocytes. The results of RNA sequencing and molecular experiments confirmed that the antipsoriatic effect of LCAA-PSF may be related to the inhibition of the activation of the TGFβ signaling pathway.

## 4. Materials and Methods

### 4.1. Chemical and Reagents

Lecigel (INCI: Lecithin and Sodium Acrylates Copolymer) was obtained from Lucas Meyer Cosmetics. Cetiol CC (INCI: dicaprylyl carbonate) was obtained from BASF Personal Care. Activonol-6 (INCI:1,2-hexanediol), Activonol-M (INCI: dipropylene glycol, hydroxyacetophenone, caprylyl glycol, dipotassium glycyrrhizinate) and Activonol-3 (INCI: propanediol) was obtained from Activon, Soline (INCI: Helianthus Annuus (sunflower)seed oil unsaponifiables) was obtained from Expanscience, and Fucocert (INCI: Biosaccharide Gum-1) was obtained from Solabia. IMQ 5% cream (TW-RX-20150930-4; iNova Pharmaceuticals) was purchased from Zuellig Pharma. IL17 rabbit polyclonal antibody (ab79056; Abcam, Cambridge, UK), FITC anti-mouse CD3εmonoclonal (553061, Clone 145-2C11; BD Biosciences, Bergen County, NJ, USA), TGFβ mouse monoclonal (3C11, sc-130348; Santa Cruz Biotechnology, Dallas, TX, USA), Ki67 (GB111499-100; Servicebio, Wuhan, China) andVEGF rabbit polyclonal (bs-1665R; BIOSS, Woburn, MA, USA) were used as the antibodies.

### 4.2. Preparation of LCAA-PSF

The materials used to prepare LCAA-PSF are listed in Table 1. Preparationwas performed as described previously [6]. To prepare LCAA-PSF, items 1 to 3 were placed in a pot and stirred evenly until smooth. Next, items 4 to 5 were mixed together in another pot. Then, this second mixture was added to the first mixture, which was then emulsified at 1200 rpm and mixed homogeneously for 15 min. Finally, items 6 to 8 were added to the above mixture and stirred evenly.

### 4.3. Animal Experiments

All experimental animals were obtained from the National Laboratory Animal Center. The animals were housed in plastic cages under controlled humidity and temperature and exposed to a 12-h/12 h light–dark cycle with free access to purified water and a standard diet. All experimental protocols were approved by the Institutional Animal Care and Use Committee of the Chang Bing Show Chwan Memorial Hospital Experimental Animal Center (approval no. 112030).

Thirty-two Balb/c mice (6–8 weeks old) were subjected to resting feeding for seven days prior to the initiation of the experiments. The mice were then randomly categorized into four groups: the control group, the IMQ group, the IMQ+LCAA group, and IMQ+LCAA-PSF group. A section of the back of each mouse was shaved (about 2 × 3 cm) prior to applying a daily (ante meridiem)topical dose of IMQ 5% cream at 62.5 mg for seven days [30]. A schematic representation of the procedure used for the animal experiments is provided in Figure 1A. Additionally, the LCAA and LCAA-PSF treatment groups were treated with 100 mg/day (post meridiem) and IMQ intervention (ante meridiem) for seven consecutive days. The levels of pigmentation, redness, texture, wrinkles, and skin elevation, and the depression volumes were monitored using Antera 3D (Miravex, Dublin, Ireland),while the level of transepidermal water loss (TEWL) was monitored using VapoMeter (Delfin Technologies, Kuopio, Finland). The Psoriasis Area and Severity Index (PASI) was employed to measure the severity grade on day 0, 2, 4, 6, and 8 [31].

### 4.4. Measurement of Blood Perfusion Changes

Blood perfusion was detected using the Laser Speckle Imaging Instrument (RFLSI III) method prior to the application of IMQ and Vaseline on each day of the experiment, as previously described [32]. The measurements obtained using this instrument aredisplayed as arbitrary perfusion units. Regions of interest (ROI) were selected according to the area of the dorsal skin that was subject to treatment: in the IMQ group, IMQ or Vaseline was used to treat the whole back; in the LCAA/LCAA-PSF group, the same area of the back was covered using Finn chambers. Data were expressed as the percentage change in the blood perfusion compared to the initial values.

### 4.5. Measurement of Dorsal Skin Thickness

Prior to the application of IMQ or Vaseline on each day of the experiments, the double-fold dorsal skin thickness was measured and averaged at two distinct sites on each mice across the experimental groups using an engineer’s micrometer (Mitutoyo, Taipei, Taiwan) with a 0.1 mm accuracy, as previously described [33]. Data were expressed as the percentage increase in the thickness of the skin on the back of the mice compared with the initial values.

### 4.6. Histology

Excised dorsal skin tissue samples were formalin-fixed (10%) and embedded in paraffin before 5 μm sections were cut and stained with hematoxylin–eosin (H&E) for further histological analysis. H&E staining was performed as previously described [34]. The scoring parameters and values were determined on the basis of the signs characteristic to psoriasis at a magnification of 200×, including the thickness of the skin epidermal layer measured by ImageJ software (Version 1.46r), and number of Munro’s microabscesses throughout the section.

### 4.7. Flow Cytometry

Spleen samples were minced through a 40 μm mesh to obtain single-cell suspensions. Cells were washed twice, and 1 × 10^6^ cells per staining were fluorescently labeled by incubation for 30 min at 4 °C with the following antibody1:100 diluted in PBS/0.2% BSA, as previously described [35]. Samples were acquired on a flow cytometer (BD Biosciences accuri C6) and analyzed using software.

### 4.8. Preparing RNA for RNA-Seq

Skin tissues were extracted from the control, IMQ, IMQ+LCAA, and IMQ+LCAAPSF groups (n = 3). Total RNA was extracted using miRNeasy Mini Kit (Qiagen, Hilden, Germany) according to the manufacturer’s recommendations and as previously described [35]. The results were then qualified using Fragment Analyzer 5200 (Agilent, Santa Clara, CA, USA) and Nano Drop (Thermo Scientific, Waltham, MA, USA). The purified RNA was used for the preparation of the sequencing library by TruSeq Stranded mRNA Library Prep Kit (Illumina, San Diego, CA, USA) following the manufacturer’s recommendations. Read 1, Sequencing Primer (5′->3′): AATGATACGGCGACCACCGAGATCTACAC; Read 2, Sequencing Primer (5′->3′): CAAGCAGAAGACGGCATACGAGAT.

### 4.9. Bioinformatic Processing for RNA-Seq

Total RNA was extracted to generate cDNA libraries for NovaSeq X plus (Illumina, San Diego, CA, USA) sequencing. Before further analyses, the relative logarithmic expression boxplots were used to control for the quality of the data. The reference genome was aligned with reference annotation (aligner: bowtie2; reference: transcript sequences), followed by gene ontology (GO) and Kyoto Encyclopedia of Genes and Genomes (KEGG) annotations with assembled differentially expressed genes (DEGs).

### 4.10. Immunohistochemistry

Immunohistochemical analysis was performed as previously described [36]. Briefly, images were obtained using a charge-coupled device camera and analyzed using the IMT i-Solution image processing, measurement, and analysis software (IMT i-Solution Inc., Winnipeg, MB, Canada).

### 4.11. Enzyme-Linked Immunosorbent Assay

The mouse serum samples were separated by centrifugation from the blood samples at 3000× *g* and 4 °C for 10 min. Then, the levels of the cytokines IL-6, IL23, TNFα, and IL17 were determined using the enzyme immunoassay kits for IL-6 (BMS607-3; Invitrogen, Waltham, MA, USA), IL23 (BMS6017; Invitrogen), TNFα (KMC0061; Invitrogen), and IL17 (BMS6001; Invitrogen), respectively. All processes were performed according to the manufacturer’s instructions.

### 4.12. Statistical Analysis

All experimental results are expressed as the mean ± standard error of the mean (SEM). One-way analysis of variance (ANOVA) with Tukey’s post hoc multiple comparison test was performed for comparisons between three or more groups. Two-way ANOVA with Bonferroni’s post hoc test was performed to compare between groups (time × percentage change or score). *p* < 0.05 was considered statistically significant.

## 5. Conclusions

**Figure 7:**
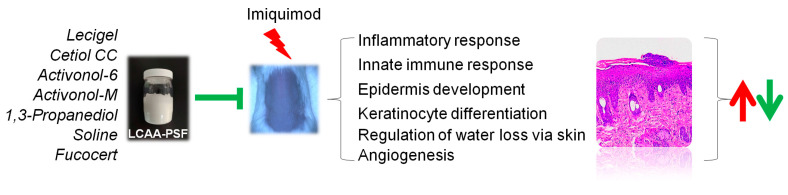
**Schematic illustration of the antipsoriatic activity of LCAA-PSF.** The red arrows indicate the promoting effects induced by IMQ, leading to increased expression or activity, whereas the green arrows denote the inhibitory effects of LCAA-PSF, resulting in decreased expression or activity.

The results indicated that LCAA-PSF exerts antipsoriatic effects in mice with IMQ-induced psoriasis. The effect was found to be related to the inhibition of inflammatory factor secretion, keratinocyte proliferation, and the activation of the TGFβ signaling pathway (Figure 7). By confirming the therapeutic effect of LCAA-PSF in the treatment of psoriasis and its corresponding mechanism, these findings provide important evidence for the clinical application of LCAA-PSF.

## Figures and Tables

**Table 1:** Information of components in LCAA-PSF.

Item	Raw Material Name	Percentage	Actual Weight (g)	Mixing Stage (I–III)
1	Pure water	75.00	225.00	I
2	1,3-Propanediol	5.00	15.00	
3	Lecigel	2.00	6.00	
4	Cetiol CC	15.00	45.00	II
5	Soline	1.00	3.00	
6	Fucocert	0.50	1.50	III
7	Activonol-6	1.00	3.00	
8	Activonol-M	0.50	1.50	

## Data Availability

Data are available upon request.

## Supplementary Materials

The following supporting information can be downloaded at: https://www.mdpi.com/article/10.3390/ijms25147720/s1.
