# Supplementary material for: Therapeutic Effect of Lecigel, Cetiol®CC, Activonol-6, Activonol-M, 1,3-Propanediol, Soline, and Fucocert® (LCAA-PSF) Treatment on Imiquimod-Induced Psoriasis-like Skin in Mice
Source: Int J Mol Sci. 2024 Jul 14;25(14):7720. doi: 10.3390/ijms25147720 (PMC11276952; doi:10.3390/ijms25147720)
Supplement: Supplementary file 1 [file ijms-25-07720-s001.zip › ijms-3071481-supplementary.pdf]

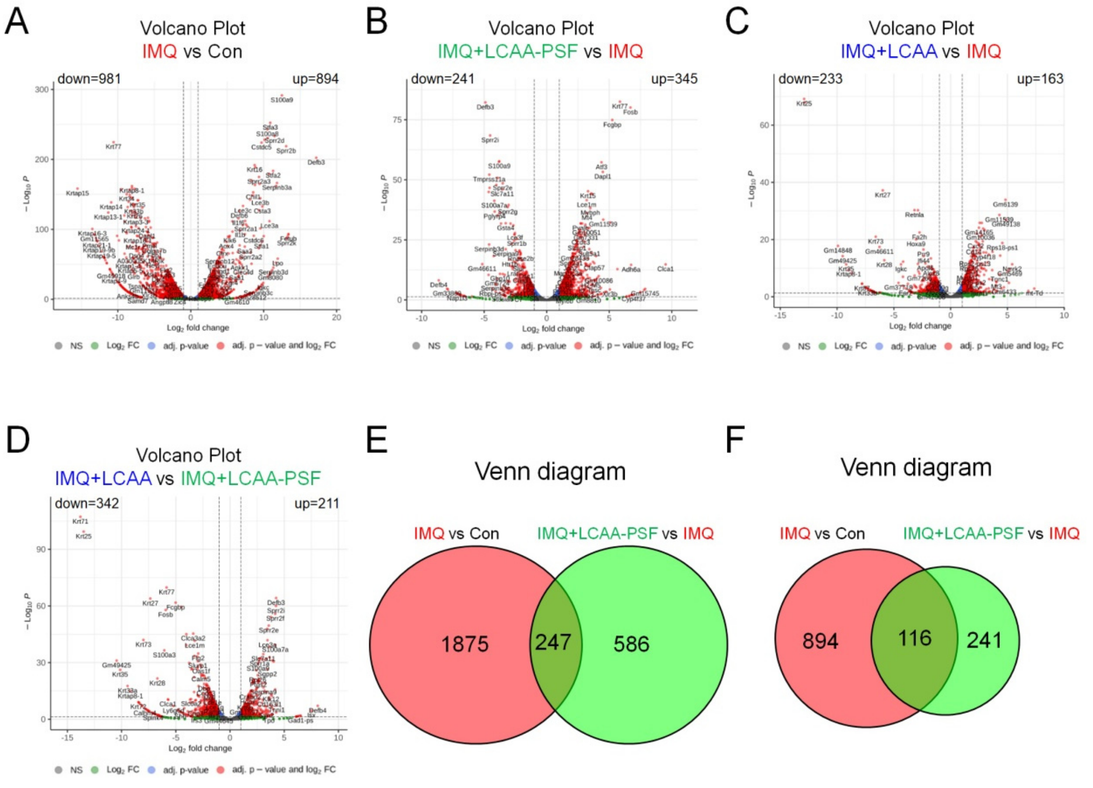

Supplemental Figure S1. Genomics of Con, IMQ, IMQ+LCAA and IMQ+LCAA-PSF on the dorsal skin.

A Volcano plot demonstrating the dual thresholds for differentially expressed gene in (A) IMQ versus Con, (B) IMQ+LCAA-PSF versus IMQ, (C) IMQ+LCAA versus IMQ and (D) IMQ+LCAA versus IMQ+LCAA-PSF. Venn diagram of the differentially expressed genes.: The number in each circle represents the amount of differentially expressed genes between the different comparisons (E) Red color: IMQ versus Con and green color: IMQ+LCAA-PSF versus IMQ. (F) Red color: IMQ versus Con increase genes and green color: IMQ+LCAA-PSF versus IMQ decrease genes. The overlapping number stands for the mutual differentially expressed genes between the different comparisons (orange color area) and the non overlapping numbers specify the genes unique to each condition.
